# Designing Vaccines to Neutralize Effective Toxin Delivery by Enterotoxigenic *Escherichia coli*

**DOI:** 10.3390/toxins6061799

**Published:** 2014-06-10

**Authors:** James M. Fleckenstein, Alaullah Sheikh

**Affiliations:** 1 Division of Infectious Diseases, Washington University School of Medicine, 660 South Euclid Avenue; Saint Louis, MO 63110, USA; 2 Molecular Microbiology and Microbiobial Pathogenesis Program, Division of Biology and Biomedical Sciences, Washington University School of Medicine, Campus Box 8051, 660 South Euclid Avenue; Saint Louis, MO 63110, USA; E-Mail: asheikh@wustl.edu; 3 Veterans Affairs Medical Center, Saint Louis, MO 63106, USA

**Keywords:** enterotoxin, diarrhea, neutralizing antibodies, heat-labile enterotoxin, heat-stable enterotoxin, enterotoxigenic *Escherichia coli*, vaccines, subunit, vaccines, live-attenuated

## Abstract

Enterotoxigenic *Escherichia coli* (ETEC) are a leading cause of diarrheal illness in developing countries. Despite the discovery of these pathogens as a cause of cholera-like diarrhea over 40 years ago, and decades of vaccine development effort, there remains no broadly protective ETEC vaccine. The discovery of new virulence proteins and an improved appreciation of the complexity of the molecular events required for effective toxin delivery may provide additional avenues to pursue in development of an effective vaccine to prevent severe diarrhea caused by these important pathogens.

## 1. Introduction

The enterotoxigenic *Escherichia coli* (ETEC) are a leading cause of diarrheal illness in developing countries where these diverse pathogens account for millions of infections and hundreds of thousands of deaths each year [1], particularly in young children under two years of age [2,3]. ETEC produce a number of different enterotoxins which either individually or collectively cause net secretion of water into the lumen of the small intestine, resulting in characteristic voluminous watery diarrhea. Illness that accompanies ETEC infection can range from mild self-limited diarrhea to severe rapid fluid losses clinically indistinguishable from cholera.

While it may be particularly important to prevent cholera-like disease to avoid deaths from diarrheal illness, the association of ETEC with delayed growth [4] and malnutrition [5,6] in developing countries could imply that an effective vaccine would have a more far-reaching impact on the health of young children at risk for these ubiquitous infections. Unfortunately, despite the global importance of these infections, and considerable investigation following the initial discovery of these organisms more than 40 years ago, there currently is no licensed broadly protective ETEC vaccine [7].

Understanding the precise molecular events involved in delivery of ETEC toxins could provide key insights that inform development of more effective vaccines. The recent identification of novel virulence factors required for optimal interaction of these organisms with target epithelial cells suggests that essential elements of toxin delivery are still being defined. Dissection of the details of ETEC pathogen-host interactions has provided additional molecules that can be targeted in new iterations of ETEC vaccines. 

## 2. ETEC Enterotoxins

The ETEC pathotype of diarrheagenic *E. coli* is defined by genes encoding one of three toxins: the heat-labile toxin (LT), and the heat-stable toxins ST-Ia (ST-P), or ST-Ib (ST-H). ETEC strains may encode any or all of these toxins each of which has been associated with severe diarrheal illness.

### 2.1. Heat-Labile Toxin (LT)

LT is an AB_5_ heterohexameric molecule that shares approximately 85% amino acid identity with cholera toxin (CT). The pentameric B subunit binds to GM-1 gangliosides on the surface of intestinal epithelial cells triggering the internalization of the catalytically active A subunit. Within the cell, the A subunit allosterically activates ADP-ribosylating factors (ARFs) which affect the ADP ribosylation of the intracellular guanine nucleotide binding protein, Gsα, abolishing its GTPase activity, and leading to constitutive activation of adenylate cyclase which increases intracellular cAMP. The resulting intracellular increases in cAMP then activate protein kinase A, in turn phosphorylating the cystic fibrosis transmembrane regulator (CFTR) [8]. It is the ensuing efflux of chloride through this channel accompanied by inhibition of Na^+^ absorption through Na/H ion exchangers (NHE3) [9] that results in the intraluminal transfer of salt and water that lead to profound diarrhea and rapid dehydration.

### 2.2. Heat-Stable Toxins (ST)

ST-Ia and ST-Ib are small (18–19 amino acid) peptides with multiple cysteine residues. These molecules are structurally similar molecular mimics of two native eukaryotic gastrointestinal peptides, guanylin and uroguanylin. Both ST-I molecules and their native homologues engage guanylyl cyclase C in the epithelial cell membrane and activate the enzyme activity leading to intracellular increases in cGMP. This cyclic nucleotide also activates protein kinases that phosphorylate and activate CFTR [10,11], resulting in toxin-induced intestinal fluid losses similar to LT.

## 3. Strategies to Neutralize Toxin Delivery

### 3.1. Essential Requirements for an Effective Vaccine

Although ETEC are defined as a pathotype by the production of the enterotoxins described above, the pathogenesis of ETEC can best be summarized as the total compilation of virulence features required for effective delivery of these toxins to their cognate receptors on the epithelial surface (Table 1). In essence then, effective ETEC vaccines need to prevent these pathogens from successfully delivering their toxin payload to the appropriate receptor either by direct neutralization of the enterotoxins and/or indirectly by engaging virulence factors that are required elements of toxin delivery.

**Table 1:** Virulence features required for optimal Enterotoxigenic *Escherichia coli* (ETEC) toxin delivery.

Virulence feature	Biology/structure	Function(s)	Reference(s)
colonization factors	plasmid-encoded fimbrial, fibrillar structures belonging to chaperone-usher-pilus (CUP) family (length ~1 µm)	structural adhesin	[12]
type 1 fimbriae	chromosomally-encoded CUP fimbrial structures (length ~1 µm)	structural adhesin	[13,14]
flagella	peritrichous arrangement; (length ~10 µm)	motility, adhesion	[15]
EtpA	170 kD secreted two-partner secretion protein	extracellular bridging adhesin	[16]
EatA	serine protease autotransporter	mucin/adhesin degradation	[17]
YghJ	secreted T2SS effector; metalloprotease	mucin degradation	[18]

### 3.2. Direct Toxin Neutralization

#### 3.2.1. Anti-LT Toxoids

Heat-labile toxin is a principal target for ETEC vaccine development. Because of inherent immunogenicity and substantial adjuvant activity [19] some form of LT is incorporated in most ETEC vaccines currently in clinical trials [20,21,22,23]. Moreover, epidemiologic studies as well as earlier vaccine studies have suggested that previous exposure to LT [24] or CT [25,26] can afford protection against subsequent infection with LT-producing ETEC. Interestingly, recent efforts to develop an LT-based ETEC vaccine patch were met with partial success in that they were protective against strains that only produced this toxin [27]. Collectively, these data support the use of LT and related molecules in ETEC vaccines; therefore, understanding the correct approach to development of LT toxoids appears to be integral to vaccine development.

Theoretically, neutralization of either the A or B subunit of LT could provide protection, either by engaging the active toxin moiety or by preventing binding of the toxin to its receptor, respectively. Indeed, experiments have demonstrated that these effects appear to be additive in preventing LT holotoxin-induced cAMP activation in target epithelial cells [16]. Mutant forms of LT have therefore been constructed [28] which are devoid of enterotoxic activity [29] but which retain the ability to stimulate antibodies against both subunits, as well as their respective contributions to LT-adjuvant activity [30].

While LT antitoxin immunity may afford some degree of protection against strains that produce only this toxin, the majority of strains produce either ST or both toxins, with approximately half of all strains producing only ST [31], where LT-toxoids would presumably offer no benefit [32]. Therefore, additional investigation will be required to determine the optimal complement of antigens that might be combined with LT-toxoids to extend coverage and enhance the overall efficacy.

#### 3.2.2. Anti-ST Toxoids

ST toxoids have faced a number of substantive challenges [33], including the small size and poor immunogenicity of these molecules, their inherent toxicity, and similarity to endogenous peptides. In addition, earlier studies suggested that high titers of antibody might be needed at the mucosal surface to neutralize activity of these toxins [34]. The ideal vaccine construct(s) would then be devoid of toxic activity, engender neutralizing immune responses to both ST-h and ST-p, and avoid cross-reactions to human uroguanylin, or guanylin. 

Studies to date have demonstrated that it is possible, either by chemical conjugation of ST peptides to carrier molecules [35,36,37,38,39], or by genetic construction of ST-fusion proteins [40,41,42,43,44,45], to induce antibodies to ST. Earlier experiments with a modified ST molecule conjugated to heat-labile toxin B subunit (LT-B) provided compelling evidence that it is possible to generate neutralizing antibodies against ST in humans. Following oral administration of the ST-LT-B conjugate, human volunteers mounted neutralizing serum and intestinal antibodies against ST [46].

Most recent attempts to generate ST antibodies have relied on genetic fusions of ST to other molecules with potent adjuvant activity such as LT. This approach has the distinct advantage of being relatively simple with respect to design and production of potential fusions permitting preclinical testing on a variety of constructs, but cannot achieve the high hapten: carrier ratios anticipated with chemical conjugation methods [33]. Nevertheless, there is compelling evidence in pigs, a natural model of ETEC infections, that this approach has merit. Fusion of ST-II to a mutant version of LT resulted in antibodies to both partners and was protective against porcine diarrheal illness in young piglets [43]. Based on these results, a similar approach is being taken with ST-I peptides relevant for humans [43,47].

A concerted effort to optimize the approach to formulating either conjugate or genetic fusions is ongoing [33,48,49]. It is likely however, that to achieve the broad-based sustained protection required for a vaccine to find utility in developing countries, additional antigens and a thorough understanding of pathogenesis will determine the best strategy to mitigate toxin delivery. 

### 3.3. Classical Colonization Factor-Based Approaches

Along with attempts to develop LT-based toxoids, plasmid-encoded fimbrial structures known as colonization factors (CFs), have been central to all efforts to develop an ETEC vaccine, including those currently in advanced stages of clinical development [7,20,21,22,23,50]. These fimbriae, which are restricted to the ETEC pathotype, are thought to be critical for colonization of the small intestine where toxin delivery is presumed to occur. Indeed, in experimental animal models of toxin delivery, antibodies against CFs have been shown to act synergistically when combined with antibodies against LT [51].

However, CF-based vaccines have faced a number of obstacles since these structures were identified [52] soon after the discovery of enterotoxigenic *Escherichia coli* in the early 1970s [53,54]. First, since the initial identification of CFA/I there have been more than 25 unique CFs identified in the global collection of ETEC to date, and ongoing DNA sequencing projects [55] suggest that new antigens will continue to be identified. This antigenic heterogeneity and lack of appreciable cross-protection have been addressed by multi-valent approaches to incorporate the most prevalent CFs [12] in candidate vaccines. Alternatively, elegant descriptions of CF biogenesis and structure have culminated in potential tip adhesin-based vaccines that do protect against diarrheal illness in an animal challenge model of ETEC infection. Many strains, as many as half of all strains in some series, however, lack any of the recognized CFs described thus far [31].

Additional data also suggest that these antigens may not alone be sufficient to stimulate the sustained robust protective responses that will likely be required of an ETEC vaccine. Epidemiologic studies of natural ETEC infections have differed with respect to whether CF immune responses correlate with protection against subsequent clinical illness in young children [24,56,57], the key target population for an ETEC vaccine. Likewise, recent studies of a live-attenuated vaccine currently in clinical development yielded suboptimal protection in a human volunteer challenge model despite robust anti-CF and anti-LT-B responses [21]. Collectively, these data suggest that our understanding of ETEC toxin delivery is presently incomplete and that additional effort may be required to optimize approaches to neutralize these critical effector molecules.

### 3.4. Identification of Novel Antigens Involved in Toxin Delivery

The challenges facing toxoid/CF-based approaches outlined above have stimulated a renewed interest in ETEC pathogenesis and fostered the identification of additional molecules that could be of importance for inclusion as subunits in a recombinant multivalent approach or targeted as part of a live-attenuated vaccine strategy. Outlined below (summarized in Table 1) are some more recently identified antigens that appear to participate in toxin delivery. 

#### 3.4.1. EtpA Two-Partner Secretion Adhesin

Interestingly, early enthusiasm for CFs as target for vaccine development arose from studies of a plasmid-cured strain of the prototypical ETEC isolate, H10407. While this strain, originally isolated from an adult with cholera-like diarrhea in Bangladesh, typically causes severe diarrhea in healthy human volunteers, recipients challenged with H10407-P, cured of a large virulence plasmid encoding CFA/I fimbriae, were virtually asymptomatic [58,59]. However, following transposon mutagenesis experiments [60] and subsequently DNA sequencing of the complete H10407 genome [61], we now understand that this particular plasmid encodes a number of additional virulence factors including ST-H, and a two-partner secretion system [60]. The ETEC “two-partner” system is actually comprised of three genes, *etpB*, an outer membrane protein responsible for transport of a secreted adhesin encoded by the *etpA* gene, and *etpC*, a putative glycosyl transferase. Similar to other TPS systems described in *Haemophilus influenza* [62], efficient secretion and stability of EtpA requires the activity of EtpC.

EtpA is in the same family of proteins as filamentous hemagglutinin (FHA), a component of modern acellular pertussis vaccines. Recognition of this similarity to a known protective antigen stimulated initial interest in further molecular characterization of EtpA following its identification in ETEC H10407. Like FHA, EtpA acts as an extracellular adhesin. EtpA acts in a unique fashion by bridging the ends of the long (10 µm) peritrichous flagella of ETEC with one or more host cell surface receptors [63]. Similar to the shorter CFs (~1 µm), EtpA-based adhesin interactions are also required for optimal delivery of heat-labile toxin as *etpA* isogenic mutants, or strains expressing EtpA with point mutations that render it incapable of interaction with flagellin (the major subunit of flagella), are demonstrably deficient in adhesion and delivery of LT to target epithelial cells [63]. Likewise antibodies against EtpA appear to act in concert with antibodies against either the A or B subunit of LT in preventing effective bacterial delivery of this toxin to epithelial cells *in vitro* [16]. Therefore, similar to the acellular pertussis vaccine approach [64], an ETEC subunit vaccine could combine multiple adhesins with a toxoid.

#### 3.4.2. Type 1 Fimbriae

While much of ETEC vaccine development effort has focused on the plasmid-encoded CFs, ETEC are also known to produce other chromosomally-encoded fimbriae known as type 1 fimbriae (T1F) [65,66]. Similar mechanisms are involved in the biogenesis of the CFs and T1F as each involves a molecular chaperone and an outer membrane usher in final assembly of the pilus structures. In contrast to the antigenic heterogeneity exhibited by the CFs however, the T1F are highly conserved with minor variations in the tip adhesin molecule FimH that may select for binding to specific sugar residues on different epithelial surfaces. Early parental vaccination studies with preparations of type 1 fimbriae (referred to as somatic fimbriae) did not afford significant protection in human volunteers challenged with the prototypical ETEC strain H10407 [67].

Interestingly however, recent transcriptome studies of ETEC [13] demonstrate that genes encoding T1F undergo significant modulation upon contact with target epithelial cells, a finding that suggests that these structures could be playing a role in bacterial adhesion. Indeed, emerging studies [14] suggest that mutants lacking either the major pilus subunit (FimA), or the tip adhesin (FimH) molecule are deficient in adhesion to target epithelial cells, and are less efficient than wild type bacteria in their delivery of heat-labile toxin.

#### 3.4.3. Flagellin

The majority of human strains of ETEC are motile and can be serotyped with H antisera [68]. Motility appears to be an absolute requirement for effective delivery of heat-labile toxin to the epithelial surface [15]. Flagellin (FliC) is the major protein subunit of flagella, with an estimated 20,000 molecules per flagellum; therefore FliC is by far the most abundant protein secreted by ETEC, and is recognized during the course of infection [69]. Each FliC molecule consists of a central variable region that accounts for H-specific serotyping, flanked by two highly conserved alpha helical regions, which promote interactions between subunits. Because ETEC encompass many H-serotypes, these antigens have been dismissed as potential vaccine candidates [68]. Nevertheless, antibodies against conserved regions of flagellin appear to protect against ETEC colonization *in vivo* [70] and impair bacterial adhesion *in vitro*, virulence features that are thought to be important prerequisites for toxin delivery.

#### 3.4.4. Secreted Mucin-Degrading Enzymes

**EatA.** The *eatA* gene, also discovered [71] on the large H10407 virulence plasmid, encodes a serine protease autotransporter protein. The 110 kDa secreted passenger domain of EatA (EatA_p_) acts in part to limit EtpA-mediated adhesion, and prevent these organisms from becoming too sticky. Somewhat counter-intuitively, *eatA* mutants are retarded in their ability to deliver LT, and antibodies directed against the passenger domain impair delivery of LT [17]. However, homologues of EatA are also present in other enteric pathogens, including *Shigella flexneri* [72], that do not make EtpA, suggesting that EatA molecules have another function. Indeed, EatA_p_ has recently been shown to cleave MUC2, the major mucin secreted by goblet cells lining the lumen of the intestine [73]. MUC2 normally serves as a major barrier to pathogen interaction with the epithelial surface. In theory, EatA and other mucin-degrading enzymes may effectively eliminate this obstacle to promote efficient delivery of LT. Interestingly, vaccination with the passenger domain EatA [18] also resulted in reduced colonization of the small intestine suggesting that this molecule is involved in optimal toxin delivery by a number of mechanisms, and that it also represents a viable vaccine candidate.

**YghJ**, an antigen initially discovered by reverse vaccinology approaches with extraintestinal pathogenic *E. coli* [74], is secreted by the same type II secretion system that is responsible for export of the heat-labile toxin from ETEC. Recent studies have demonstrated that YghJ is recognized by convalescent antibody following ETEC infection [69]. YghJ belongs to a larger family of secreted metalloproteases capable of degrading intestinal mucins [18]. YghJ accelerates delivery of LT both *in vitro* and *in vivo*, and antibodies directed at YghJ impair effective toxin delivery suggesting that this highly conserved antigen common to most ETEC strains examined to date could be a valuable target for vaccine development along with pathotype-specific mucin-degrading proteins like EatA.

## 4. Antigen Conservation

One potential impediment to effective ETEC vaccine development is the underlying plasticity of *E. coli* genomes in general [75]. As noted above, this genomic plasticity has complicated traditional CF-based approaches to ETEC vaccines and necessitated incorporation of multiple CFs to achieve broad coverage of prevailing ETEC strains circulating in a given geographic area. While no completely conserved virulence molecule specific to the ETEC pathotype has been described to date, emerging data do suggest that EtpA and EatA may be more highly conserved than the classical vaccine targets and are present in as many as 70% of strains described to date [76,77]. The chromosomally-encoded features common to many *E. coli*, such as YghJ and the T1F tend to be more highly conserved. The degree to which these can be targeted without impacting commensal strains of *E. coli* has not been determined.

## 5. Conclusions

The enterotoxigenic *Escherichia coli* continue to present a formidable challenge to attempts to develop a broadly protective vaccine. However, ETEC vaccines to date have focused on a relatively small subset of antigens that are not sufficiently conserved or do not alone offer sufficient protective efficacy to provide broad-based protection from severe diarrhea caused by ETEC. An improved understanding of the molecular events involved in ETEC interactions with the intestinal epithelium that are required for efficient delivery of its enterotoxins has provided additional targets for interdiction that can complement existing strategies as well as emerging toxoid-based approaches to toxin neutralization. These novel targets and an abundance of genomic, and immuno-proteomic data on the horizon should inform a rational approach to designing next generation broadly protective ETEC vaccines.
